# Universal Germline Testing of Unselected Cancer Patients Detects Pathogenic Variants Missed by Standard Guidelines without Increasing Healthcare Costs

**DOI:** 10.3390/cancers13225612

**Published:** 2021-11-10

**Authors:** Adrienne T. Perkins, Derrick Haslem, Jessica Goldsberry, Katherine Shortt, Laura Sittig, Sharanya Raghunath, Christopher Giauque, Shawnee Snow, Gail Fulde, Bryce Moulton, David Jones, Lincoln Nadauld

**Affiliations:** Intermountain Healthcare, Precision Genomics, Saint George, UT 84790, USA; adrienne.perkins@imail.org (A.T.P.); derrick.haslem@imail.org (D.H.); jessica.goldsberry@imail.org (J.G.); katherine.shortt@imail.org (K.S.); laura.sittig@imail.org (L.S.); sharanya.raghunath@imail.org (S.R.); chris.giauque@imail.org (C.G.); shawnee.snow@imail.org (S.S.); gail.fulde@imail.org (G.F.); bryce.moulton@imail.org (B.M.); david.jones2@imail.org (D.J.)

**Keywords:** hereditary cancer, healthcare costs, precision oncology, pathogenic germline variants, germline testing

## Abstract

**Simple Summary:**

Clinical genetic testing likely underestimates the frequency of pathogenic germline variants (PGVs) in a cancer patient population due to strict qualifications designated by practice guidelines. Accurate ascertainment of PGVs in cancer patients can be paramount to the treatment of hereditary cancer syndromes. Our prospective study aimed to (1) elucidate PGV frequency in an unselected cohort of cancer patients by offering universal germline testing regardless of eligibility by clinical guidelines and to (2) evaluate the impact of receiving such testing on cost of care. Results confirmed that over 50% of patients who harbored a PGV would not have qualified for testing under current guidelines and there was no increase in healthcare costs for patients who tested positive for a PGV. It is therefore feasible to offer universal germline testing for cancer patients in the clinic to detect PGVs without increasing healthcare costs.

**Abstract:**

Purpose: To accurately ascertain the frequency of pathogenic germline variants (PGVs) in a pan-cancer patient population with universal genetic testing and to assess the economic impact of receiving genetic testing on healthcare costs. Methods: In this prospective study, germline genetic testing using a 105-gene panel was administered to an unselected pan-cancer patient population irrespective of eligibility by current guidelines. Financial records of subjects were analyzed to assess the effect of PGV detection on cost of care one year from the date of testing. Results: A total of 284 patients participated in this study, of which 44 patients (15%) tested positive for a PGV in 14 different cancer types. Of the patients with PGVs, 23 patients (52%) were ineligible for testing by current guidelines. Identification of a PGV did not increase cost of care. Conclusion: Implementation of universal genetic testing for cancer patients in the clinic, beyond that specified by current guidelines, is necessary to accurately assess and treat hereditary cancer syndromes and does not increase healthcare costs.

## 1. Introduction

Inherited germline cancer susceptibility genes are estimated to play a role in roughly 4–24% of all cancers diagnosed [1,2,3,4,5,6,7,8,9,10,11,12,13,14,15,16,17,18,19,20]. Detection of hereditary cancer syndromes through clinical genetic testing can impact treatment, improve patient outcomes, and lower healthcare costs for proband patients. Eligibility to receive clinical genetic testing is determined by a patient’s cancer type, age at diagnosis, personal and family history of cancer, and other criteria outlined by current guidelines [21,22,23,24]. However, recent studies have challenged the guidelines by offering universal testing to cancer patients irrespective of whether they qualified for genetic testing. Such studies carried out in cancer patients with selected cancer types, like breast [1,7,19], kidney [4], pancreatic [2,15], prostate [16], and colorectal cancer [20,25], as well as pan-cancer patient populations either selected for advanced stage [11] or unselected [10,13,14] found increased detection of pathogenic or likely-pathogenic germline variants (PGVs) in patients not eligible to receive testing. While this work provides support for universal genetic testing of cancer patient populations, there is less evidence on the impact of such testing on cost of care.

The use of genetic testing to guide targeted cancer treatment, known as precision oncology, leads to improved patient outcomes without increasing healthcare costs [26,27]. To this end, our study aimed to evaluate the ability of practice guidelines to accurately ascertain cancer patients with PGVs and the economic impact of genetic test results on cost of care for proband patients.

## 2. Materials and Methods

### 2.1. Patient Cohort

This was a prospective study of newly presenting, unselected, solid tumor cancer patients 18 years of age or older, who were recruited from practices at Intermountain Healthcare in St. George and Cedar City, Utah, from 4 June 2019, to 18 December 2020. Patients with hematological malignancies or previously identified hereditary cancer syndromes were excluded from the study. A total of 284 patients were enrolled in the study. Clinical research coordinators identified and recruited the patients from lists of clinic visits. The study was approved by the Intermountain Healthcare institutional review board and all participants provided written informed consent. Upon enrollment, a buccal swab was collected, and samples were tested for germline variants using a 105 gene panel in the CLIA-certified, CAP-accredited clinical lab at Intermountain Precision Genomics. A positive test result denoted the presence of a PGV. Variants of unknown significance were not disclosed to patients and were thus reported as negative test results. Patient test results were reviewed and disclosed to the patients by their providing oncologist. Patients with positive test results were referred to a certified genetic counselor. Personal and family history information from requisition forms and medical records was reviewed manually and deidentified before analysis.

### 2.2. Patient Eligibility for Clinical Genetic Testing and Family History

Patients with positive test results were scored as either eligible or ineligible for clinical genetic testing according to the National Comprehensive Cancer Network (NCCN) [21,22,24], and the American College of Medical Genetics (ACMG) and National Society of Genetic Counselors (NSGC) [23] guidelines for their respective cancer type. Patients with PGVs were categorized for family history based on whether they claimed a family history of cancer (yes), denied a family history of cancer (no), or if their family history was not mentioned or unknown due to adoption (unknown).

### 2.3. Cost of Care Analysis

Strata software was used to join financial and clinical data to allocate costs down to charge activity and summarize costs by medical record number (MRN) and patient encounters. All encounters across the entire Intermountain system within the study time frame were pulled and summed to provide a comprehensive total cost for each patient within one year from the date of the genetic test. The one-year time frame was chosen to improve accuracy of the analysis by limiting the chance that patients switched insurers during that time.

### 2.4. Panel Composition

A comprehensive 105 gene version of the in-house Intermountain Precision Genomics Inhera Cancer Panel (Supplementary Table S1) was used to sequence patients. This 105 gene panel was created by addition of 77 genes to the original Inhera panel. Sequencing used a capture probe design originally developed by the Alberta Precision Laboratories, Molecular Genetics Laboratory at the University of Alberta Hospital, Edmonton, Alberta. One hundred and five genes implicated in inherited cancer risk were sequenced by next-generation sequencing (NGS). Copy number variants (CNVs) in a subset of 26 genes were tested for and confirmed by a combination of NGS and MLPA analysis (Supplementary Table S1).

### 2.5. Sequencing and Germline Variant Calling

DNA was extracted in the Precision Genomics CAP-certified clinical laboratory from DNA Genotek ORAcollect buccal swab samples using the Promega Maxwell RSC Whole Blood DNA kit on the Promega Maxwell RSC system. Libraries were prepared using Illumina TruSight and/or Nextera Flex reagents and sequenced using the Illumina Miseq and Nextseq platforms to at least 100× of 2 × 150 bp.

Resultant sequence data were demultiplexed using bcl2fastq [28] and aligned to the reference human genome sequence GrCh37 using BWA-MEM v0.7.17 [29]. Sequencing quality was assessed using Picard v2.18.21+ [30] and FastP v0.20.0 [31]. Indel variant and single-nucleotide variants (SNVs) were called using the Genome Analysis Tool Kit (GATK v4.0.12+) [32] and copy number variants (CNVs) were called using DECoN v1.0.2 [33]. The genetic test was validated to detect single nucleotide polymorphisms (SNPs), and insertions and deletions (Indels) up to 20 bp in length and copy number variants (CNVs) of at least one exon in length. Variants >20 bp and less than one exon in length may have been detected with reduced sensitivity. CNVs were confirmed by MLPA and SNVs and indels with coverage <30× or <30% minor allele frequency were confirmed by Sanger sequencing.

### 2.6. Variant Interpretation 

Fabric Enterprise was used to perform variant classification using ACMG/AMP guidelines [34] with modifications according to current ClinGen recommendations (www.clinicalgenome.org/curation-activities/variant-pathogenicity/documents/) last accessed 11 April 2021.

### 2.7. Statistical Analyses

All data are presented as descriptive statistics. A two-tailed t-test was used to compare all continuous variables. A *p*-value of ≤0.05 was considered statistically significant. 

## 3. Results

### 3.1. Characterization of Patient Cohort 

The patient cohort analyzed in this study consisted of 284 patients (mean age [SD], 66.6 [12.5] years; 128 [45%] male). Cancer stage at diagnosis was as follows: 73 patients (26%) with stage 0/I disease, 57 patients (20%) with stage II disease, 70 patients (25%) with stage III disease, 77 patients (27%) with stage IV disease, and the disease stage was missing for 7 patients (2%). Breast (80 patients, 28%), colon (41 patients, 14%), prostate (23 patients, 8%), lung (23 patients, 8%), and pancreatic (20 patients, 7%) cancer were the most common primary cancers diagnosed in the population. Race/ethnicity was reported as American Indian/Alaskan Native (1 [0.4%]), Asian (2 [0.7%]), African American (1 [0.4%]), White/Hispanic (5 [1.8%]), White/Not Hispanic (266 [93.7%]), and declined/unavailable (9 [3.2%]). 

### 3.2. Patients with Pathogenic/Likely-Pathogenic Germline Variants

Of the 284 patients in this study, 44 patients (15%; mean age [SD], 66 [12.2] years; 16 [36%] male) tested positive for a pathogenic or likely-pathogenic germline variant (PGV) on the comprehensive 105 gene panel (Supplementary Table S2). Two patients each harbored two PGVs yielding a total of 46 PGVs in the dataset. Cancer stage at diagnosis in patients with PGVs was as follows: 13 patients (28%) with stage 0/I disease, 8 patients (18%) with stage II disease, 11 patients (25%) with stage III disease, 10 patients (23%) with stage IV disease, and the disease stage was missing for 2 patients (5%) (Supplementary Table S2). Breast (12 patients, 27%), colon (6 patients, 14%), and lung (6 patients, 14%) cancer were the most common primary cancers diagnosed among patients with PGVs out of 14 different cancer types total (Supplementary Table S2). A portion of 25 patients (57%), of the 44 patients with PGVs, claimed a family history of cancer, 11 patients (25%) denied a family history of cancer, and for 8 patients (18%) their family history of cancer was unknown (Supplementary Table S2). Patients with PGVs identified as Asian (1 [2%]), White/Not Hispanic (41 [93%]), and declined/unavailable (2 [5%]). 

### 3.3. Pathogenic/Likely-Pathogenic Germline Variants

**Figure 1:**
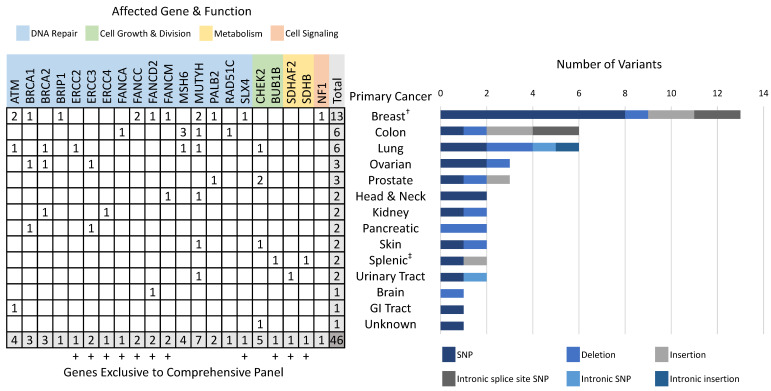
Characterization of gene variants, gene function, genes exclusive to the comprehensive panel, and variant type by primary cancer diagnosis in patients with PGVs. All patients were heterozygous for all variants. Data represents 44 patients, 46 variants. SNP = single nucleotide polymorphism. ^†^, one patient harbored two PGVs (BRIP1 SNP and NF1 insertion). ^‡^, one patient harbored two PGVs (SDHB insertion and BUB1B SNP).

Forty-four patients harbored a total of 46 PGVs found in genes on both the original Inhera and the comprehensive panel, in variants of different types detected by analysis, and primarily in genes in the DNA repair pathway (Supplementary Table S2). The most commonly affected genes were *MUTYH* (7 [15.2%]), *CHEK2* (5 [10.9%]), *ATM* (4 [8.7%]), *MSH6* (4 [8.7%]), *BRCA1* (3 [6.5%]), and *BRCA2* (3 [6.5%]) (Figure 1). One female breast cancer patient tested positive for PGVs in both *BRIP1* and *NF1*, while one patient with splenic cancer harbored PGVs in both *SDHB* and *BUB1B* (Figure 1). All patients were heterozygous for all variants, including those harboring variants in *MUTYH* for which the monoallelic variant has an unclear risk of cancer. Of the 44 patients with PGVs, 14 patients (32%) tested positive for variants in genes found exclusively on the comprehensive 105 gene panel and would have been missed using the smaller Inhera panel (Figure 1). SNPs were the most common variant (22 [48%]) followed by deletions (11 [24%]), insertions (6 [13%]), intronic splice site SNPs (4 [9%]), intronic SNPs (2 [4%]), and intronic insertions (1 [2%]) (Figure 1). The overwhelming majority of variants (37 [81%]) were detected in genes involved in DNA repair (Figure 1) which have important implications for intervention with targeted therapy. Fewer variants were detected in genes with molecular functions such as cell growth and division (6 [13%]), metabolism (2 [4%]), and cell signaling (1 [2%]) (Figure 1). 

### 3.4. Eligibility for Clinical Genetic Testing in Patients with PGVs

**Figure 2:**
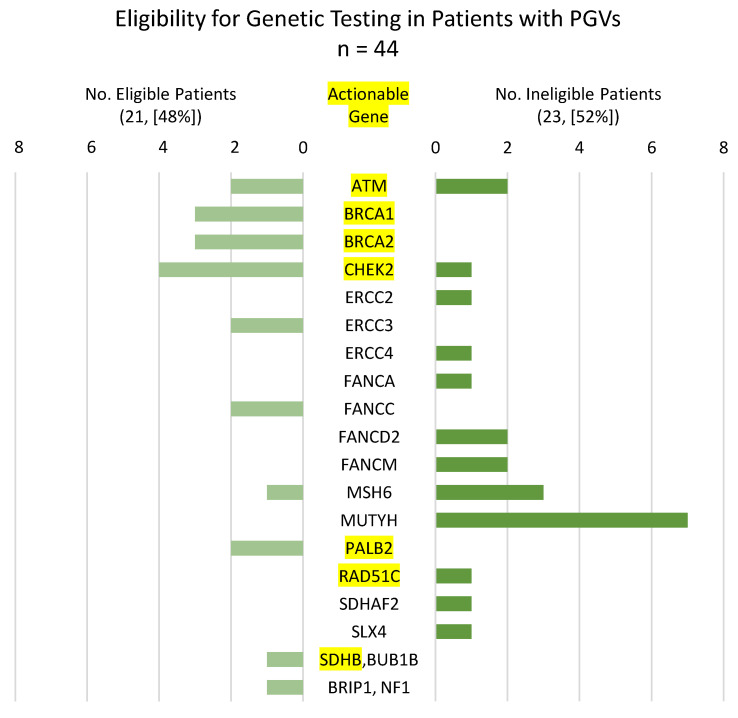
Number of patients with PGVs eligible to receive clinical genetic testing according to NCCN, ACMG, and NSGC guidelines for cancer type. Data represent 46 variants from 44 patients. Two patients harbored more than one variant and are listed at the bottom of the figure. Yellow highlighting indicates clinically significant genes.

When the 44 patients with PGVs were scored for eligibility to receive genetic testing based on the most current NCCN, ACMG, and NSGC guidelines for cancer type [21,22,23,24], over half (23 [52%]) were found to be ineligible (Figure 2, Supplementary Table S2). The majority of these ineligible patients were breast, colon, and lung cancer patients which were the top three cancer diagnoses in this study (Supplementary Table S2). Of these ineligible patients, 12 (27%) reported a family history of cancer while 9 patients (20%) denied a family history of cancer, and for 2 patients (5%) their family history was unknown (Supplementary Table S2). All patients with heterozygous variants in *MUTYH* were found to be ineligible, a finding in line with the unclear risk for this variant in hereditary cancer. Twenty-five patients (57%) with PGVs had a variant in one of the ACMG 59 genes that are clinically actionable and for which published clinical management and surveillance recommendations exist (Figure 2). Fourteen patients (32%) with variants in clinically actionable genes had a family history of cancer, while six (14%) did not, and in five patients (11%) it was unknown (Supplementary Table S2). Patients who were eligible for genetic testing were more likely to harbor clinically actionable PGVs (17 actionable, 4 not actionable) than those who were ineligible (8 actionable, 15 not actionable) and vice versa (Figure 2). Nevertheless, over one-third (8, [35%]) of ineligible patients tested positive for a clinically actionable PGV. The two patients (4.5%) with PGVs in actionable genes that are exclusive to the comprehensive panel tested positive for variants in *SDHAF2* and *SDHB* (Supplementary Table S2).

### 3.5. Cost of Care Analysis in Patients with PGVs 

**Figure 3:**
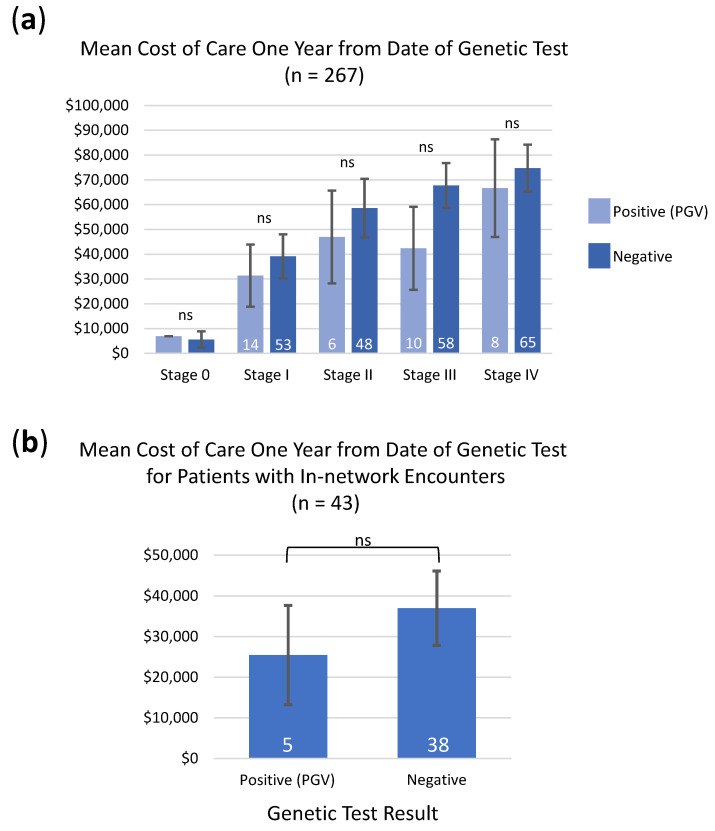
Mean cost of care within one year from the date patients received genetic testing. (**a**) Data from patients with one year follow-up broken down by stage at diagnosis. Stage 0 positive *n* = 1; stage 0 negative *n* = 4. (**b**) Data for in-network encounters. Error bars represent standard error. *p*-values based on student’s t-test. n.s = not significant. N-values indicated within each bar.

Examination of healthcare-associated cost data for patients with one year follow-up from the date of genetic testing (*n* = 267) revealed that cost of care did not increase for patients with PGVs regardless of the stage of cancer at diagnosis (Figure 3a). For patients with disease stage I–IV, detection of a PGV was on average associated with a lower cost of care (Figure 3a). When cost of care was examined for a subset of in-network encounters for patients who were likely to receive all their care through Intermountain (*n* = 43), the same trend held in that there was no increase in cost of care for patients who harbored PGVs (Figure 3b). Overall, cost of care does increase as stage of disease at diagnosis increases (Figure 3a). 

## 4. Discussion

In this study, universal genetic testing of a pan-cancer patient population revealed that 15% of patients (44 of 284) carried a PGV in a cancer susceptibility gene, and that over half (23 of 44) of those with PGVs failed to meet current guidelines for clinical genetic testing. Contrary to industry opinions, there was not a measurable difference in cost of care between patients with positive and negative test results. 

Fifteen percent of patients in this study had a hereditary cancer syndrome designated by an inherited PGV, of which 2.5% (7 of 284) were heterozygous for *MUTYH* and have an unclear cancer risk. The current literature dictates a range of PGV detection between approximately 4–24% in cancer patients depending upon the type of cancer [1,2,3,4,5,6,7,8,9,10,11,12,13,14,15,16,17,18,19,20]. Our data agrees with similar studies [10,13,14] conducted in an unselected cancer patient population with universal testing where a range of 5–14% of patients tested positive for PGVs. The range of detection likely reflects the wide range of number of patients included in the studies, the number of genes on the testing panels, the types of cancer present in the populations, or differences in the frequency of PGVs at the population level. One advantage of large multi-gene panels is the ability to capture PGVs in patients who harbor more than one PGV. Additionally, large gene panels help to ascertain PGVs in patients with cancers not typically associated with a specific PGV. While it is not clear that such PGVs are responsible for the observed malignancy, this represents an opportunity for further investigation including functional validation studies in model organisms. Conversely, known associations between PGVs and genetically predisposed cancer types does not preclude patients from developing additional cancer types that could have been identified through standard screening practices. Altogether, our study demonstrated that universal genetic testing is feasible in the cancer clinic using a large 105 gene panel. 

The most impactful result from our study was that 52% of patients with PGVs would not have been eligible to receive genetic testing based on current practice guidelines. This result concurs with former studies [1,2,4,7,10,11,13,14,15] where 5–70% of patients with PGVs did not qualify for genetic testing based on the guidelines for their respective cancer type. Again, the wide range in the percentage of patients who did not meet guidelines is likely representative of the number of genes included on the panels. In our study, patients who claimed or denied a family history of cancer were found to be in both the eligible and ineligible categories. Furthermore, in some cases, family history and eligibility for testing were revealed after the initial identification of the PGV when the patient met with a genetic counselor. This demonstrates that self-reported family history of cancer can be inaccurate and on its own is a poor predictor of genetic testing eligibility. Implementation of widespread universal testing would eliminate issues with collecting family history in the clinic, a significant component on which guidelines are based, that is not at the forefront of most providers minds. The fact that in our study there were cancer patients with clinically actionable mutations that failed to meet testing guidelines (35%), regardless of whether or not a family history of cancer was reported, further underscores the importance of offering testing to all cancer patients to ensure the best treatment options. Collectively, these findings emphasize the constraints of the current guidelines to accurately ascertain the prevalence of hereditary cancer in a cancer patient population and argue for ubiquitous genetic testing of cancer patients in the clinic.

Evaluation of the economic impact of genetic testing within one year from the test date found no increase in healthcare costs for patients with PGVs. While the cost of care does increase with stage of cancer at diagnosis, our data suggest that patients who harbor a PGV may experience lower healthcare costs on average. Our work extends the findings in the field that stress the necessity of universal genetic testing by demonstrating that detection of a PGV does not put additional financial burden on the patient. Additionally, these results suggest that healthcare costs could be reduced if hereditary cancer is detected at early disease stages, which underlines the value of cascade testing. 

The limitations of this study include the fact that the study only enrolled cancer patients who presented to clinics in southern Utah and therefore consisted of a total of 284 subjects. The analyses may be biased by the small size of this cohort as well as the lack of diversity as the vast majority of participants identified as White/Not Hispanic. This limits the application of the findings to other regions of the United States. Moreover, there was no follow-up to assess long-term outcomes in patients that may have been conferred by change in care management.

One advantage of conducting research at an integrated healthcare system is the access to study participants’ financial data which allowed us to gauge the economic impact of genetic test results on cost of care within one year of testing. To our knowledge, this is the first study to demonstrate that identification of an inherited PGV does not increase healthcare costs in a population of pan-cancer patients who received universal genetic testing. Our study demonstrates that it is feasible and valuable from both a financial and medical standpoint to offer universal genetic testing to all patients with a new cancer diagnosis. 

Our study is novel because it establishes the feasibility in terms of human and technological resources for universal genetic testing in a community health system as compared to an academic health setting. There was no need to add nurse navigators, laboratory technicians, genetic counselors, primary care providers, or additional resources to meet study needs. All patient test results were managed through the EMR and stored in the EMR for future reference. Our methodology is therefore applicable and transferrable not only to other clinics within Intermountain Healthcare, but also to external multi-specialty cancer clinics. 

## 5. Conclusions

In this study where genetic testing was blindly offered to solid tumor cancer patients not selected for by traditional criteria, PGVs were identified in more patients than would have been detected under standard practice guidelines. Given that detection of a PGV does not raise cost of care within one year of testing, our data support the adoption of widespread multi-gene panel germline genetic testing into oncology clinical care models. In the long term, our work provides additional evidence for genetic testing at the population level which will unlock the ultimate potential of precision medicine to prevent cancer, improve outcomes, and lower healthcare costs in disease free individuals.

## Data Availability

The data that support the findings of this study are available on request from the corresponding author L.N. The data are not publicly available due to them containing information that could compromise research participant privacy/consent.

## Supplementary Materials

The following are available online at https://www.mdpi.com/article/10.3390/cancers13225612/s1, Table S1. Genes included in genetic test and examples can be found at http://www.mdpi.com/2076-2615/6/6/40/htm (accessed on 15 October 2021). Table S2. Characterization of patients identified to harbor pathogenic germline variants.
