# Supplementary material for: Universal Germline Testing of Unselected Cancer Patients Detects Pathogenic Variants Missed by Standard Guidelines without Increasing Healthcare Costs
Source: Cancers (Basel). 2021 Nov 10;13(22):5612. doi: 10.3390/cancers13225612 (PMC8616044; doi:10.3390/cancers13225612)
Supplement: Supplementary file 1 [file cancers-13-05612-s001.zip › cancers-1420779-supplementary.pdf]

cancers

MDPI

Universal Germline Testing of Unselected Cancer Patients Detects Pathogenic var Iants Missed by Standard Guidelines without Increasing Healthcare Costs

Adrienne T. Perkins, Derrick Haslem, Jessica Goldsberry, Katherine Shortt, Laura Sittig, Sharanya Raghunath, Christopher Giauque, Shawnee Snow, Gail Fulde, Bryce Moulton, David Jones and Lincoln Nadauld

Table S1. Genes included in genetic test.

**Comprehensive 105 Gene Hereditary Cancer Panel**							
Inhera Panels		Genes Exclusively on Comprehensive Panel					
APC	MUTYH	AIP	DIS3L2	FANCG	NF2	SBDS	XPA
ATM	NBN	ALK	EGFR	FANCI	NSD1	SDHA	XPC
BARD1	NF1	BAP1	ERCC2	FANCL	PDE4D	SDHAF2	
BLM	PALB2	BUB1B	ERCC3	FANCM	PHOX2B	SDHB	
BMPR1A	PMS2	CASR	ERCC4	FH	PMS1	SDHC	
BRCA1	POLD1	CDC73	ERCC5	FLCN	POLE	SDHD	
BRCA2	PTEN	CDK4	EXT1	GATA2	PPM1D	SLX4	
BRIP1	RAD50	CDKN1B	EXT2	GNAS	PRF1	SMARCB1	
CDH1	RAD51C	CDKN1C	EZH2	GPC3	PRKAR1A	SUFU	
CHEK2	RAD51D	CDKN2A	FANCA	HNF1A	PTCH1	TMEM127	
EPCAM	SMAD4	CEBPA	FANCB	HRAS	RB1	TSC1	
MLH1	STK11	CEP57	FANCC	KIT	RECQL4	TSC2	
MRE11A	TP53	CYLD	FANCD2	MAX	RET	VHL	
MSH2		DDB2	FANCE	MEN1	RHBDF2	WRN	
MSH6		DICER1	FANCF	MET	RUNX1	WT1	

Bold text indicates genes analyzed for CNVs.

Table S2. Characterization of patients identified to harbor pathogenic germline variants (PGV).

Patient #	Sex	Age at Dx	Diagnosis	Stage	Family History of Cancer	Testing Eligibility	Gene with PGV[a]	Variant Type[b]	P/LP[c]	Gene Exclusive to Expanded Panel	Clinically Actionable	Gene Function
1	Female	55	Brain Cancer	Stage IV	No	Ineligible	FANCD2	deletion	LP	Yes	No	DNA Repair
2	Female	53	Breast Cancer	Stage 0	Yes	Ineligible	ATM	SNP	P	No	Actionable	DNA Repair
3	Female	71	Breast Cancer	Stage I	Yes	Eligible	PALB2	insertion	P	No	Actionable	DNA Repair
4	Female	75	Breast Cancer	Stage IA	Yes	Eligible	BRIP1, NF1	SNP, insertion	P, P	No, No	Actionable, Actionable	DNA Repair, Cell Signaling
5	Female	45	Breast Cancer	Stage IA	Unknown	Eligible	FANCC	SNP	P	Yes	No	DNA Repair
6	Female	52	Breast Cancer	Stage IA	Yes	Eligible	ATM	SNP	LP	No	Actionable	DNA Repair
7	Female	63	Breast Cancer	Stage IA	Yes	Ineligible	MUTYH	SNP	P	No	No	DNA Repair
8	Female	51	Breast Cancer	Stage IA	No	Ineligible	SLX4	SNP	P	Yes	No	DNA Repair
9	Female	67	Breast Cancer	Stage IA	Yes	Ineligible	FANCM	SNP	P	Yes	No	DNA Repair
10	Female	60	Breast Cancer	Stage IIA	Yes	Eligible	FANCC	intronic splice site SNP	P	Yes	No	DNA Repair
11	Female	80	Breast Cancer	Stage IIA	Yes	Ineligible	FANCD2	intronic splice site SNP	P	Yes	No	DNA Repair
12	Female	65	Breast Cancer	Stage IIA	Yes	Ineligible	MUTYH	SNP	P	No	No	DNA Repair
13	Female	31	Breast Cancer	Stage IIIC	No	Eligible	BRCA1	deletion	P	No	Actionable	DNA Repair

14	Female	64	Cancer of unknown primary	Stage IV	Unknown	Eligible	CHEK2	SNP	LP	No	Actionable	Cell Growth & Division
15	Female	60	Colon Cancer	Stage I	No	Ineligible	MSH6	SNP	P	No	Actionable	DNA Repair
16	Male	44	Colon Cancer	Stage I	Unknown	Ineligible	MUTYH	intronic splice site SNP	P	No	No	DNA Repair
17	Male	92	Colon Cancer	Stage II	No	Ineligible	FANCA	deletion	P	Yes	No	DNA Repair
18	Female	66	Colon Cancer	Stage IIA	Yes	Eligible	MSH6	insertion	LP	No	Actionable	DNA Repair
19	Male	70	Colon Cancer	Stage IIIB	No	Ineligible	MSH6	insertion	P	No	Actionable	DNA Repair
20	Male	64	Colon Cancer	Stage IV	Yes	Ineligible	RAD51C	intronic SNP	P	No	Actionable	DNA Repair
21	Female	59	GI Tract Cancer	Stage III	Yes	Ineligible	ATM	SNP	P	No	Actionable	DNA Repair
22	Female	58	Head & Neck Cancer	Stage I	No	Ineligible	FANCM	SNP	P	Yes	No	DNA Repair
23	Male	63	Head & Neck Cancer	Stage I	Yes	Ineligible	MUTYH	SNP	P	No	No	DNA Repair
24	Female	70	Kidney Cancer	Stage III	Yes	Ineligible	ERCC4	SNP	LP	Yes	No	DNA Repair
25	Male	77	Kidney Cancer	Stage IV	Yes	Eligible	BRCA2	deletion	LP	No	Actionable	DNA Repair
26	Female	72	Lung Cancer	Stage III	Yes	Ineligible	MUTYH	SNP	P	No	No	DNA Repair
27	Male	66	Lung Cancer	Stage III	Unknown	Ineligible	CHEK2	deletion	P	No	Actionable	Cell Growth & Division
28	Female	59	Lung Cancer	Stage IIIA	Unknown	Eligible	BRCA2	deletion	P	No	Actionable	DNA Repair
29	Female	80	Lung Cancer	Stage IIIB	No	Ineligible	ERCC2	SNP	P	Yes	No	DNA Repair
30	Female	69	Lung Cancer	Stage IV	Yes	Eligible	ATM	intronic SNP	P	No	Actionable	DNA Repair
31	Male	81	Lung Cancer	Stage IV	No	Ineligible	MSH6	intronic insertion	P	No	Actionable	DNA Repair
32	Female	73	Ovarian Cancer	Stage III	Unknown	Eligible	ERCC3	SNP	P	Yes	No	DNA Repair
33	Female	51	Ovarian Cancer	Stage III	Yes	Eligible	BRCA2	deletion	LP	No	Actionable	DNA Repair
34	Female	56	Ovarian Cancer	unknown	Yes	Eligible	BRCA1	SNP	P	No	Actionable	DNA Repair
35	Female	82	Pancreatic Cancer	Stage IA	Yes	Eligible	ERCC3	deletion	LP	Yes	No	DNA Repair
36	Female	69	Pancreatic Cancer	Stage IIB	No	Eligible	BRCA1	deletion	P	No	Actionable	DNA Repair
37	Male	82	Prostate Cancer	Stage IIIC	Yes	Eligible	CHEK2	SNP	LP	No	Actionable	Cell Growth & Division
38	Male	77	Prostate Cancer	Stage IV	Yes	Eligible	CHEK2	deletion	P	No	Actionable	Cell Growth & Division
39	Male	79	Prostate Cancer	unknown	Unknown	Eligible	PALB2	insertion	P	No	Actionable	DNA Repair
40	Male	69	Skin Cancer	Stage IV	Yes	Eligible	CHEK2	deletion	P	No	Actionable	Cell Growth & Division
41	Male	70	Skin Cancer	Stage IV	Yes	Ineligible	MUTYH	SNP	P	No	No	DNA Repair
42	Male	55	Splenic Cancer	Stage II	Unknown	Eligible	SDHB, BUB1B	insertion, SNP	P, P	Yes, Yes	Actionable, No	Metabolism, Cell Growth & Division
43	Male	88	Urinary Tract Cancer	Stage II	No	Ineligible	SDHAF2	intronic SNP	LP	Yes	Actionable	Metabolism
44	Male	69	Urinary Tract Cancer	Stage IV	Yes	Ineligible	MUTYH	SNP	P	No	No	DNA Repair

[a] All patients were heterozygous for all variants; [b] SNP = single nucleotide polymorphism; [c] P/LP = pathogenic, likely-pathogenic.
